# The multicellular interplay of microglia in health and disease: lessons from leukodystrophy

**DOI:** 10.1242/dmm.048925

**Published:** 2021-07-20

**Authors:** Woutje M. Berdowski, Leslie E. Sanderson, Tjakko J. van Ham

**Affiliations:** Department of Clinical Genetics, Erasmus MC University Medical Center, PO Box 2040, 3000 CA, Rotterdam, The Netherlands

**Keywords:** Microglia, Myelination, Leukodystrophy, Oligodendrocytes, Astrocytes, Genetic disease

## Abstract

Microglia are highly dynamic cells crucial for developing and maintaining lifelong brain function and health through their many interactions with essentially all cellular components of the central nervous system. The frequent connection of microglia to leukodystrophies, genetic disorders of the white matter, has highlighted their involvement in the maintenance of white matter integrity. However, the mechanisms that underlie their putative roles in these processes remain largely uncharacterized. Microglia have also been gaining attention as possible therapeutic targets for many neurological conditions, increasing the demand to understand their broad spectrum of functions and the impact of their dysregulation. In this Review, we compare the pathological features of two groups of genetic leukodystrophies: those in which microglial dysfunction holds a central role, termed ‘microgliopathies’, and those in which lysosomal or peroxisomal defects are considered to be the primary driver. The latter are suspected to have notable microglia involvement, as some affected individuals benefit from microglia-replenishing therapy. Based on overlapping pathology, we discuss multiple ways through which aberrant microglia could lead to white matter defects and brain dysfunction. We propose that the study of leukodystrophies, and their extensively multicellular pathology, will benefit from complementing analyses of human patient material with the examination of cellular dynamics *in vivo* using animal models, such as zebrafish. Together, this will yield important insight into the cell biological mechanisms of microglial impact in the central nervous system, particularly in the development and maintenance of myelin, that will facilitate the development of new, and refinement of existing, therapeutic options for a range of brain diseases.

## Abstract (precis)

**Summary:** Microglia abnormalities are increasingly linked to white matter diseases. Complementing analyses of leukodystrophy patient material with animal models yields insight into the impact and therapeutic potential of microglia across diverse brain disorders.

## Introduction

Macrophages are myeloid-derived cells distributed throughout the vertebrate body that have tissue-specific subsets and functions based on their origin and tissue of residence (Varol et al., 2015; Wynn et al., 2013; Guilliams et al., 2020). Their functions extend far beyond immunological roles, contributing to processes ranging from organogenesis, tissue repair and lipid metabolism to electrical conductivity in the heart, and their organ-specific regulatory functions are thought to affect virtually every vertebrate organ (Gordon and Martinez-Pomares, 2017; Pollard, 2009). Multiple macrophage subtypes exist within the central nervous system (CNS), including microglia, as well as perivascular, meningeal and choroid plexus macrophages (Li and Barres, 2018), each possibly varying in function in accordance with its respective tissue niche (Guilliams et al., 2020). CNS macrophages exist within a complex microenvironment of neuronal and non-neuronal cells, including astrocytes and oligodendrocyte lineage cells, with which they interact extensively through a variety of direct and indirect means (Box 1, Glial cells: The Pillars of White Matter Integrity). Owing to their more clearly recognized relevance for brain function and more extensive characterization, this Review will primarily focus on microglia. Microglia originate from myeloid progenitor cells in the yolk sac that colonize the brain during early embryonic development (Herbomel et al., 1999; Ginhoux et al., 2010). Whether these yolk sac macrophages are the only source of microglia in the adult brain is still debated (Xu et al., 2015; Ferrero et al., 2018, 2020; Sheng et al., 2015; De et al., 2018). Under homeostatic conditions, microglia are long-lived and can proliferate locally, with limited peripheral contribution (Mildner et al., 2007; Sheng et al., 2015; Ajami et al., 2018). Therefore, owing to the relative isolation and enduring nature of microglia compared to many other macrophages, the effects of microglial aberrations are arguably more penetrant, or may accumulate more significantly, than might be expected in other tissues.

Until the early 2000s, the roles of microglia in the healthy brain were perceived almost exclusively as immune regulation and phagocytic scavenging. Mainly owing to improved real-time *in vivo* imaging in zebrafish and mouse models, microglia were subsequently found to be highly dynamic in ways previously not imagined. By extending and retracting their ramified processes, microglia constantly scavenge, monitor and modulate the brain environment (Nimmerjahn et al., 2005; Davalos et al., 2005; Peri and Nüsslein-Volhard, 2008; Wake et al., 2009; Tremblay et al., 2010; Hughes and Appel, 2020). For example, microglia directly shape brain development by pruning synapses (Stevens et al., 2007; Paolicelli et al., 2011; Schafer et al., 2012) and myelin sheaths (Hughes and Appel, 2020), and by clearing apoptotic neurons and myelin debris after damage, which is crucial for remyelination after injury (Neumann et al., 2009; Lampron et al., 2015; Cignarella et al., 2020). They also regulate neuronal activity by direct interactions with neuronal somata (Tremblay et al., 2010; Cserép et al., 2020; Badimon et al., 2020; Li et al., 2012), and sculpt the extracellular matrix to promote synapse formation (Nguyen et al., 2020). In fact, it is now widely agreed that microglia interact with essentially all cellular components of the CNS and have vital, yet insufficiently understood, roles in shaping brain development and maintaining normal brain function and integrity (Li and Barres, 2018). For a brief summary of the known interactions of microglia with other cells in the CNS, see Box 1. Of particular interest are their recent connections to the development and maintenance of white matter – the heavily myelinated tissue of the CNS that facilitates connectivity throughout the brain (Box 1), and is particularly damaged in genetic disorders known as leukodystrophies. However, the mechanisms that underlie the putative roles of microglia in promoting myelin health or disease remain largely unknown.

**Box 1. Glial cells: The Pillars of White Matter Integrity**

The CNS grey matter contains mostly neuronal cell bodies that process chemo-electrical signals via axonal signaling through the white matter, enabling rapid connectivity between neurons and between brain regions. The white matter, which comprises as much as ∼60% of the brain volume in humans, is mainly composed of glial cells, capillaries and myelinated axons. Myelination is more plastic and dynamic throughout life than previously thought, and neuronal activity-dependent adaptive myelination plays a role in learning and memory (Williamson and Lyons, 2018, Mount and Monje, 2017). Neuronal activity, even that caused by epileptic seizures, can in fact induce myelination (Gibson et al., 2014, Mensch et al., 2015, Marisca et al., 2020, Hughes and Appel, 2020). Microglia and astrocytes interact with and regulate the membrane processes of oligodendrocytes, thereby shaping, maintaining and repairing myelin (reviewed by Baydyuk et al., 2020, Traiffort et al., 2020, de Waard and Bugiani, 2020). Therefore, to thoroughly understand the functions of microglia in myelination and myelin health, one should examine their dynamic interactions with oligodendrocytes and astrocytes.

**Oligodendrocytes and myelin:** oligodendrocytes are the myelin-producing cells of the brain, synthesizing large amounts of membrane and wrapping it around the axons in multiple compacted layers in an incredibly complex origami-like manner. Mature myelinating oligodendrocytes arise from OPCs (Bergles and Richardson, 2015). As there are many more OPCs present in the CNS throughout development and adult life than one might expect based on the number of myelinating oligodendrocytes, it has been suggested that OPCs may serve additional undiscovered purposes in the brain (Marisca et al., 2020). Myelin contains ∼40% water, whereas the remaining dry mass consists of a high proportion of lipids (∼70%) and a lower proportion of proteins (∼30%), among which myelin basic protein (MBP) accounts for 22-35% (Jahn et al., 2009). A main property of myelin is that it facilitates conduction by insulating the axon and enabling the action potential to jump from one node of Ranvier to the next, thereby greatly speeding up signal propagation. Additionally, myelin protects axons and can metabolically support them by providing carbon-based metabolites, such as lactate and pyruvate (Aggarwal et al., 2011, Philips and Rothstein, 2017).

Under homeostatic conditions, microglia modulate OPC survival and differentiation (Hagemeyer et al., 2017, Wlodarczyk et al., 2017); for example, via insulin-like growth factor (IGF1) (Ye et al., 2002, Wlodarczyk et al., 2017). Upon myelin injury, microglia can promote OPC differentiation and, consequently, remyelination (Miron et al., 2013). Additionally, microglia clear myelin debris after white matter damage, which is crucial for the remyelination process (Lampron et al., 2015, Cantuti-Castelvetri et al., 2018, Miron, 2017).

**Astrocytes:** Astrocytes are the most abundant cell lineage in the adult vertebrate brain, outnumbering neurons by a factor of nine in humans (Kimelberg and Nedergaard, 2010). Astrocytes are a heterogeneous cell population characterized by extended fiber-like protrusions forming a dense network. Processes surrounding the synaptic cleft are necessary for the uptake and release of neurotransmitters, including glutamate, and can regulate synapse formation and refinement (Haydon and Carmignoto, 2006, Allen and Eroglu, 2017). A subset of astrocytes forms perivascular end-feet that enwrap the capillaries and contribute to the BBB, where they can regulate blood flow and BBB integrity, and shuttle nutrients, such as glucose from the blood to neurons and other brain cells, including oligodendrocytes (Kimelberg and Nedergaard, 2010, Haydon and Carmignoto, 2006). Astrocytes in a ‘reactive’ state are associated with a broad spectrum of neurological diseases and can be either beneficial or detrimental to disease progression. This state and associated nomenclature has been recently defined by Escartin et al. (2021).

Astrocytes can secrete cytokines and growth factors that influence myelination by regulating OPC survival, oligodendrocyte differentiation and maturation (Lanciotti et al., 2013, Lundgaard et al., 2014, Baydyuk et al., 2020, Traiffort et al., 2020). Besides communication via soluble factors, direct cell-cell contacts in the form of gap junctions, formed by connexins, provide oligodendrocytes, but also neurons and axons, with lipids and glucose as both an energy supply and as substrates for myelination (Orthmann-Murphy et al., 2008, Camargo et al., 2017). As all glial cells interact, modulate and regulate one another on many levels, including signaling, metabolism and cell development, a holistic cellular view is needed to comprehend the diseased and healthy brain, especially regarding myelination and white matter integrity.

**Box 2. The Zebrafish – *In Vivo* Modelling of Glial Cell Dynamics and Myelination in Genetic Brain Disorders**

Zebrafish (*Danio rerio*) are especially suitable for studying early brain development and pediatric disease: the embryos develop rapidly, *ex utero*, are transparent, and it is now generally accepted that all the major glial cells known in mammals (Box 1) are also present in zebrafish. Importantly, they share extensive conservation of function, morphology and gene expression (Lyons and Talbot, 2014; Marisca et al., 2020; Hughes and Appel, 2019; Oosterhof et al., 2017; Mu et al., 2019; Chen et al., 2020). Cellular dynamics and interactions, including processes such as myelination and myelin maintenance, can be studied non-invasively with high spatiotemporal precision. With over 70% of genes shared with humans and carrying orthologs of ∼82% of human disease-associated genes, and continuously improving genetic tools, the zebrafish is an increasingly popular model for investigating genetic disorders (Howe et al., 2013). For more information, please see extensive reviews by Duncan et al. (2011), Ackerman and Monk (2016) and Rutherford and Hamilton (2019) on zebrafish and rodent models of leukodystrophies and myelination.

Transgenic zebrafish lines expressing fluorescent proteins under the control of specific promoters can be used to visualize cell populations in real time (Preston and Macklin, 2015). This allows cell type-specific *in vivo* monitoring of, for example, the calcium signaling of neurons and astrocytes (Akerboom et al., 2012; Mu et al., 2019), cell-cell interactions, cell dynamics and intracellular processes, such as phagocytosis (van Ham et al., 2014). Zebrafish allow real-time non-invasive imaging of cells in their natural environment in a whole organism. By imaging fish on consecutive days, researchers can easily follow individual cells longitudinally. This also allows for an unbiased approach to the discovery of novel cell biological processes. Recent examples of this are the discovery of microglial regulation of neuronal activity (Li et al., 2012), pruning of myelin sheaths during development (Hughes and Appel, 2020), regulation of myelination by neuronal activity via synaptic vesicle release (Mensch et al., 2015) and the existence of functionally distinct subsets of oligodendrocytes in the spinal cord (Marisca et al., 2020).

Zebrafish have a tremendous regenerative capacity after injury, which can be exploited to identify molecules and pathways that regulate myelin and axonal regeneration, which could, for example, aid in treatment strategies for spinal cord injury (Cigliola et al., 2020). Drug screening can be performed rapidly, on a large scale and *in vivo*, which is a major advantage compared to murine models (MacRae and Peterson, 2015). Drug screens in zebrafish have another unexpected benefit: as drugs are screened in a complete organism, any leads discovered in zebrafish are more likely to be effective in additional *in vivo* systems. Indeed, several drugs discovered only recently in zebrafish have made it to phase 2 clinical trials [e.g. Dravet syndrome (Baraban et al., 2013)]. Another drug screen in an *abcd1^−/−^* zebrafish model shows the potential for leukodystrophies, revealing metabolic rerouting of saturated to mono-unsaturated VLCFAs following pharmacologically increased expression of the enzyme SCD1 (Raas et al., 2021). This led to fewer behavioral abnormalities and reduced lipid toxicity, making it a promising therapeutic strategy for X-ALD and other peroxisomal diseases.

## Microglia and disease

### Current microglia-targeting therapies

Neuropathological, epidemiological and human genetic studies provide strong evidence that microglia play a modifying role in a wide variety of congenital and sporadic brain diseases (Graeber and Stre'rt, 1990; Mattiace et al., 1990; Perry et al., 2010; Prinz and Priller, 2014; Ulland and Colonna, 2018), and could thus represent a universal treatment target. Indeed, even though the precise roles of microglia in health and disease are incompletely understood, existing data on how hematopoietic stem cell transplantation (HSCT) therapy – a means of supplementing the endogenous myeloid cell repertoire – affects the diseased human brain strongly support this direction. HSCT provides donor myeloid cells that can migrate into the brain, likely resulting in a renewed population of healthy macrophages/microglia in the diseased CNS (Wolf et al., 2020; Bennett et al., 2018). Transplantations have been shown to promote remyelination in several leukodystrophies (Musolino et al., 2014; Krägeloh-Mann et al., 2013; Boucher et al., 2015; van Egmond et al., 2013; Cartier and Aubourg, 2010; Weinstock et al., 2020; Mochel et al., 2019; Gelfand et al., 2020), suggesting that replenishing either the abundance or function of microglia can have unprecedented benefits for a range of severe brain diseases. Inversely, transient microglial depletion by clodronate liposomes or chemical inhibitors of colony stimulating factor 1 receptor (CSF1R), a key regulator of macrophage development, chemotaxis and survival (Stanley and Chtu, 2014), can ameliorate disease progression in mouse models of neurodegenerative diseases, such as Alzheimer's disease by facilitating remyelination and alleviating symptoms of myelination defects (Casali et al., 2020; Hansen et al., 2018; Janova et al., 2018; Lloyd et al., 2019; Spangenberg and Green, 2017; Spangenberg et al., 2019).

### Microglia as key players in the pathology of leukodystrophy

Leukodystrophies constitute a heterogeneous group of rare genetic disorders characterized by primary involvement of the CNS white matter and neuropathology involving glial cells (van der Knaap et al., 2019). The name ‘leukodystrophy’ is derived from Greek and literally translates to abnormal growth of the white matter. Although individual leukodystrophies are rare, the overall leukodystrophy incidence is around 1 in 7600 live births (Bonkowsky et al., 2010). The majority of leukodystrophies initiate in childhood and most affected individuals fail to reach adulthood. Cognitive and motor decline are the most common clinical symptoms, but dementia, personality changes and depression are also highly prevalent (Table 1).

**Table 1.**
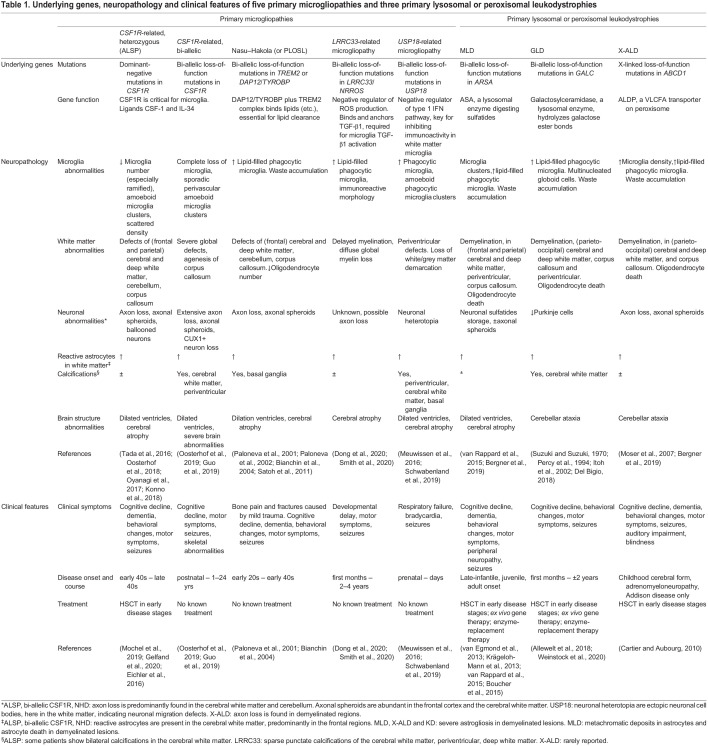
Underlying genes, neuropathology and clinical features of five primary microgliopathies and three primary lysosomal or peroxisomal leukodystrophies

We will compare the cellular pathology of two groups of leukodystrophies to build a case for a more global involvement of microglia in neuropathology and, in doing so, highlight the broad promise of this glial cell type as a therapeutic target. First, we will describe microgliopathies, which are white matter diseases believed to be caused primarily by microglial abnormalities (Prinz and Priller, 2014), including *CSF1R*-related leukodystrophy, Nasu–Hakola disease (NHD) related to recessive genetic variants in *TREM2* and *DAP12/TYROBP*, and leukodystrophies related to recessive genetic variants in the *LRRC33* (also known as *NRROS*) or *USP18* genes. Next, we will address lysosomal and peroxisomal leukodystrophies, including metachromatic leukodystrophy (MLD), X-linked adrenoleukodystrophy (X-ALD) and globoid cell leukodystrophy (GLD), also known as Krabbe disease, that all show beneficial responses to HSCT. Based on the overlapping pathological features of these two groups, we will describe possible mechanisms, supported by circumstantial evidence, through which aberrant microglia could lead to white matter abnormalities and overall disturbance of brain health. We argue that studying these diseases provides a unique opportunity to elucidate the putative roles of microglia in modulating myelin development and maintenance, while expanding our understanding of the detrimental effects caused by aberrant microglia in the human brain.

## Primary microgliopathies: leukodystrophies with central microglial pathology

**Fig. 1.**
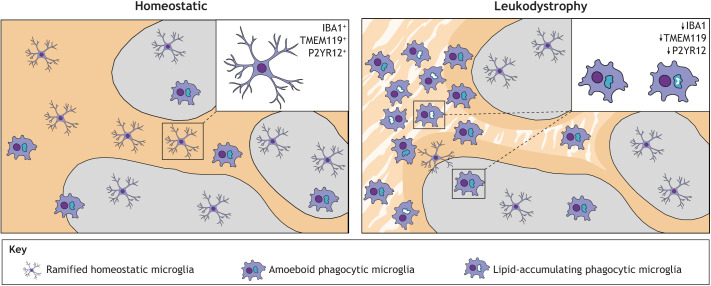
**Schematic representation of microglial phenotypes in the grey and white matter of homeostatic and leukodystrophic cortical tissue.** In the homeostatic brain, most microglia are evenly distributed in the white and grey matter, and appear ramified, expressing homeostatic markers, such as IBA1, TMEM119 and P2YR12. In the leukodystrophic brain, the white matter is affected by degenerative lesions (striped pattern) and microglia are unevenly distributed, clustering in certain areas, especially within white matter lesions. Phagocytic microglia are abundant within lesions and present an amoeboid shape with large CD68^+^ intracellular lysosomal vacuoles. Near leukodystrophic lesions, phagocytes display lipid accumulation. Ramified microglia are localized to the grey matter, where their declining density towards the white matter lesions correlates with a gradual loss of homeostatic gene expression and gain of lysosomal CD68 expression. Grey, grey matter; yellow, white matter; stripe pattern, degenerative white matter lesions.

Glial cell pathology is a major hallmark of leukodystrophies (Garcia et al., 2020). A classification system based on the main glial contributor to the underlying pathogenesis was recently proposed (Vanderver et al., 2015; van der Knaap and Bugiani, 2017). Microgliopathies comprise disorders caused by mutations in genes predominantly expressed by microglia, ultimately leading, either directly or indirectly, to white matter defects (Prinz and Priller, 2014). Leukodystrophies related to bi-allelic loss-of-function mutations in *CSF1R*, *TREM2*, *TYROBP*, *LRRC33*/*NRROS* or *USP18* can be categorized as primary microgliopathies (Oosterhof et al., 2019; Meuwissen et al., 2016; Schwabenland et al., 2019; Dong et al., 2020; Smith et al., 2020). To date, only *CSF1R* also demonstrates a dominant form of disease (Rademakers et al., 2011). These genetic disorders lead to overlapping microglial phenotypes,including disturbed distribution, gene expression and morphology (Fig. 1), which we discuss in more detail in the coming sections. Individuals affected by these genetic disorders present with severe white matter degeneration predominantly in the frontal and parietal lobes, in the corpus callosum and in periventricular regions. Axon pathology, dilated ventricles and cerebral atrophy are also highly prevalent, although the U-fibers are mostly spared. Additionally, most affected individuals present with calcifications, predominantly periventricular, although these occur specifically in the basal ganglia of NHD and *USP18*-related disease. An overview of clinical and pathological features can be found in Table 1.

### CSF1R-related leukodystrophy

**Fig. 2.**
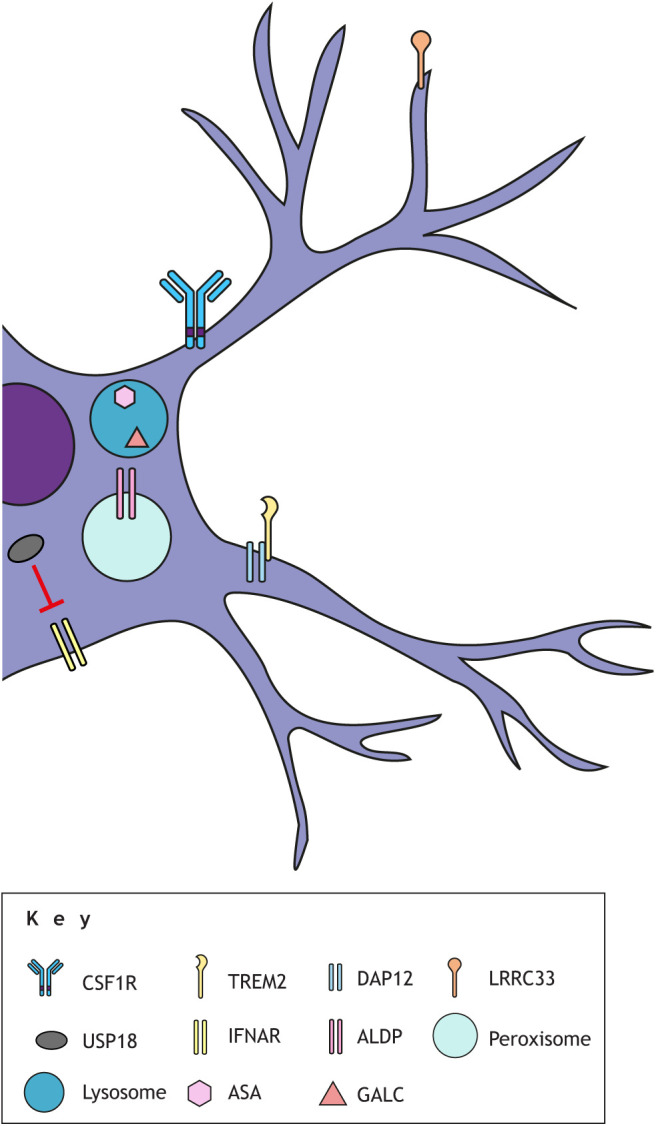
**Leukodystrophy-associated proteins in microglia.** Several microglial proteins, either at the cell surface or residing within organelles, are involved in leukodystrophy-related cellular processes. CSF1R is a dimeric transmembrane receptor that is a key regulator of microglia and macrophage biology, including proliferation, migration and survival, and binds ligands CSF-1 and IL-34. TREM2 is a single-pass transmembrane receptor involved in lipid metabolism and phagocytic clearance by macrophages and microglia. It binds anionic ligands, including phospholipids and bacterial components, and forms a signaling complex with DAP12, which is a dimeric transmembrane adapter required for the cell-surface expression of TREM2, forming a signaling complex. LRRC33 is a leucine-rich repeat-containing protein that anchors latent TGF-β1 at the cell surface. It is required for activation of the TGF-β1 pathway in macrophages and microglia. The USP18 isopeptidase binds to the intracellular domain of IFNAR2, thereby negatively regulating the IFN-I pathway. ALDP, a peroxisomal ABC half-transporter encoded by *ABCD1*, transports VLCFAs. ASA, encoded by *ARSA*, is a lysosomal enzyme that digests sulfatides, glycosphingolipids that are highly enriched in myelin. GALC, encoded by *GALC*, is a lysosomal enzyme catabolizing galactosylceramide and psychosine, both major glycosphingolipids in myelin. ASA and GALC are not known to be expressed at high levels in macrophages and microglia, but there are strong indications that their functions are required by microglia in the context of myelin.

As mentioned above, CSF1R is a key regulator of microglia and macrophage biology *in vivo*, regulating aspects of proliferation, migration and survival (Herbomel et al., 2001; Dai et al., 2002; Erblich et al., 2011; Oosterhof et al., 2018; Pridans et al., 2018; Rojo et al., 2019; Kuil et al., 2019b, 2020) (Fig. 2). It is a transmembrane receptor tyrosine kinase primarily expressed on mononuclear phagocytic cells (Stanley and Chitu, 2014; Hume et al., 2020). It functions as a homodimer, binding dimers of either CSF-1 or IL-34 (Stanley and Chitu, 2014). In humans, heterozygous mutations in the *CSF1R* gene can lead to adult-onset leukoencephalopathy with axonal spheroids and pigmented glia (ALSP; Table 1) (Rademakers et al., 2011). Loss of CSF1R in zebrafish, mice and rats results in a severe depletion of most macrophages and microglia, though a small number of amoeboid IBA1^+^ cells remain (Oosterhof et al., 2018; Erblich et al., 2011; Pridans et al., 2018). *Csf1r^−/−^* mice show apparently normal myelination but have reduced oligodendrocyte numbers and rarely survive to adulthood (Erblich et al., 2011). *Csf1r*^−/−^ rats have reduced myelination (Pridans et al., 2018), and *csf1r*-deficient zebrafish have transient myelin abnormalities, as shown in a recent preprint (Djannatian et al., 2021). Unlike mice, both *Csf1r*-deficient rats and zebrafish survive to adulthood.

The first records of ALSP date from 1936, when it was described as pigmentary orthochromatic leukodystrophy (POLD) and later as hereditary diffuse leukoencephalopathy with axonal spheroids (HDLS) (Konno et al., 2018). Following the identification of their common genetic cause, they are now considered to be the same entity (Nicholson et al., 2013; Adams et al., 2018). Besides global white matter abnormalities and neuropathological features (Table 1), a major hallmark of ALSP is the presence of axonal spheroids, which contain organelles and aggregated proteins, such as neurofilament and amyloid precursor protein (APP) (Lin et al., 2010). Human postmortem histological studies showed that cells expressing IBA1 (also known as AIF1), a protein highly enriched in microglia and macrophages, and P2YR12, a microglia-specific protein, are reduced (Tada et al., 2016; Oosterhof et al., 2018) and have a decreased density with a scattered distribution in three cortical areas, both in white and grey matter (Oosterhof et al., 2018). Dense clusters of amoeboid microglia, which are associated with increased phagocytic activity, are nevertheless also observed in cortical areas, predominantly in the cerebral white matter and the corpus callosum, and these cells are strongly positive for the lysosomal protein CD68 (Oosterhof et al., 2018; Tada et al., 2016; Oyanagi et al., 2017; Kempthorne et al., 2020). CD68 is a widely used marker for visualizing phagocytes, including macrophages and microglia. Numerous lipid-laden microglia are present within degenerated white matter lesions, indicating myelin phagocytosis. Our own group recently reported that in brain tissue from a patient with bi-allelic loss-of-function *CSF1R* mutations, microglia are completely absent, except for sporadic perivascular clusters of phagocytic CD68^+^ cells (Oosterhof et al., 2019). The developmental appearance of amoeboid IBA1^+^ and CD68^+^ cells in subcortical regions, including the corpus callosum and periventricular regions (Ling and Wong, 1993), a phenomenon first described by del Rio-Hortega (1932), is likely connected to a developmental phagocytic role of these cells. Our group's experiments with zebrafish further showed that induced cell death in the brain of partial *csf1r*-deficient zebrafish causes a local increase in microglia numbers mainly by migration, which could further promote the local depletion of microglia in other brain areas, as observed in patients (Oosterhof et al., 2018). Altogether, these findings strongly suggest that microglial depletion or loss of homeostatic microglia, which may precede the onset of symptoms by many years, may be a key pathogenic initiating event in *CSF1R*-related leukodystrophy.

However, it is possible that some brain pathology may be an indirect consequence of abnormalities caused by macrophage deficiency outside of the CNS. For example, skeletal abnormalities resulting from defective osteoclasts extend to the skull bones, as seen in individuals with bi-allelic *CSF1R* variants and reminiscent of those observed in *Csf1r*-deficient zebrafish, mouse and rat models, which may additionally affect brain development (Oosterhof et al., 2019; Guo et al., 2019; Caetano-Lopes et al., 2020; Pridans et al., 2018). Furthermore, *Csf1r*-deficient rats exhibit a loss of liver macrophages and disrupted liver function, including dysregulation of lipid metabolism and of the GH/IGF1 system, which could be rescued by bone marrow transplantation (Keshvari et al., 2020). Meanwhile, specific depletion of microglia by knockout of a *Csf1r* enhancer in mice, where other brain-resident macrophages remain, results in less severe myelin and brain abnormalities than those seen in *Csf1r^−/−^* mice (Rojo et al., 2019). This suggests that macrophages beyond microglia may indirectly contribute to the severe neuropathology seen in CSF1R deficiency.

The strong effect of heterozygous mutations in *CSF1R*, resulting in severe progressive leukodystrophy and dramatically reduced microglia numbers, can be explained by a dominant-negative effect: all but a few causative genetic variants in ALSP patients are missense and affect one of the two tyrosine kinase domains (Konno et al., 2017). *In vitro*, mutant receptors can still dimerize with wild-type receptors and likely perturb their function (Hume et al., 2020; Pridans et al., 2013). Therefore, a single missense variant likely leads to a loss of function closer to 75% rather than 50%. In line with this, in several instances of reported bi-allelic loss-of-function variants, including a premature stop codon, no typical ALSP signs were reported in parents that were heterozygous for one of the variants (Oosterhof et al., 2019; Guo et al., 2019; Monies et al., 2017).

The ability to deplete microglia via CSF1R inhibition, based on mouse model studies, was recently recognized as a possible therapeutic strategy for the treatment of several brain diseases associated with inflammation, including Alzheimer's disease (Spangenberg and Green, 2017; Spangenberg et al., 2019; Casali et al., 2020; Hansen et al., 2018) and glioma (Akkari et al., 2020). In ALSP, it might be expected that providing healthy myeloid donor cells, with normal CSF1R signaling, via HSCT during an early stage of disease could provide beneficial effects, possibly by boosting the microglia pool. This is indeed the case in mice (Bennett et al., 2018). Additionally, multiple publications have reported cases in which HSCT delayed or even halted disease progression in ALSP patients (Mochel et al., 2019; Gelfand et al., 2020; Eichler et al., 2016). Importantly, whether individuals benefit from HSCT is likely highly dependent on the level of disease progression at the start of treatment and the ability of an individual to cope with the effects of myeloablative treatment.

Thus, findings in CSF1R-related leukodystrophy patients, with heterozygous and bi-allelic variants, and in CSF1R-mutant animal models strongly indicate that a loss of microglia and macrophages in development and/or in adult life strongly affects both myelin and overall brain health, and that these effects can be at least partially abrogated through HSCT.

### Nasu–Hakola disease

NHD, or polycystic lipomembranous osteodysplasia with sclerosing leukoencephalopathy (PLOSL), was described in the early 1970s by Hakola (1972) and Nasu et al. (1973). Over 200 cases have been reported in the literature since then. Unique hallmarks of this disease are bone pain and fractures caused by mild trauma and bilateral basal ganglial calcifications (Paloneva et al., 2001). Genetic analyses identified causative recessive loss-of-function mutations in one of two genes: triggering receptor expressed on myeloid cells 2 (*TREM2*) or DNAX activating protein of 12 kDa (*DAP12*), also known as TYRO protein kinase-binding protein (*TYROBP*) (Paloneva et al., 2002) (Fig. 2). TREM2 is a transmembrane receptor and DAP12/TYROBP a transmembrane adaptor protein that together form a signaling complex (Deczkowska et al., 2020; Ulland and Colonna, 2018). Both TREM2 and DAP12 are highly expressed in myeloid cells, including microglia. Thus, taken together with the CNS abnormalities and pathology (Table 1), NHD is considered a microgliopathy (Bianchin et al., 2004; Xing et al., 2015; Konishi and Kiyama, 2018; Bakker et al., 2000; Schmid et al., 2002). Ligands for TREM2 include lipids and bacterial components, including the Alzheimer's disease-associated ApoE and Aβ oligomers (Konishi and Kiyama, 2018). TREM2 in particular has been extensively studied in mouse and induced pluripotent stem cell (iPSC) models, mainly due to its link to microglial involvement in Alzheimer's disease pathology (reviewed by Ulland and Colonna, 2018). TREM2 is involved in lipid metabolism and TREM2^+^ lipid-associated macrophages in mouse adipose tissue can sense aberrations in lipid composition and respond by driving the expression of genes associated with phagocytosis, lipid catabolism and energy metabolism (Jaitin et al., 2019). Microglia in *Trem2*^−/−^ mice fail to upregulate these processes in response to acute demyelination (Poliani et al., 2015; Cantoni et al., 2015). TREM2-deficient microglia can phagocytize myelin debris after demyelination but are unable to break it down, especially cholesterol, resulting in intracellular lipid accumulation (Cantoni et al., 2015; Linnartz-Gerlach et al., 2019; Nugent et al., 2020). Hence, TREM2 signaling appears to be a major pathway by which macrophages respond to changes in tissue-level lipid homeostasis, and lack of TREM2 signaling contributes to ineffective responses to myelin injury and turnover.

In genetic mouse models of NHD, microglia numbers vary depending on the age of the animal and the brain region analysed. In *Trem2*^−/−^ or *Dap12*^−/−^ mice, microglia numbers are typically reduced during early development but differences from wild-type mice are largely absent at later stages (Filipello et al., 2018; Poliani et al., 2015; Nataf et al., 2005; Otero et al., 2009; Kaifu et al., 2003). Loss of functional TREM2 has also been shown to impair the phagocytic capacity of microglia in a range of model systems, including mutant mice (Filipello et al., 2018; Poliani et al., 2015), iPSC-derived microglia (Garcia-Reitboeck et al., 2018; McQuade et al., 2020) and in primary mouse microglia (Takahashi et al., 2005; Hsieh et al., 2009; Kleinberger et al., 2014). Mouse models recapitulate several pathological features seen in patients (Table 1), including degeneration of the white matter (Kaifu et al., 2003; Nataf et al., 2005), bone abnormalities (Kaifu et al., 2003; Nataf et al., 2005) and axonal pathology (Cantoni et al., 2015). Importantly, a loss of microglia or macrophages is also not found in postmortem brain samples of NHD patients (Oyanagi et al., 2017). However, an increased phagocytic phenotype and intracellular waste accumulation in both ramified (Satoh et al., 2011) and CD68^+^ amoeboid microglia and macrophages is observed in the white matter (Paloneva et al., 2001; Oyanagi et al., 2017), which is consistent with defective processing of phagocytized material.

### *LRRC33/NRROS* and *USP18*-related leukodystrophies

Two severe pediatric neurodevelopmental white matter diseases caused by bi-allelic variants in the microglia-associated genes *LRRC33/NRROS* or *USP18* have recently been described*.* The pathological features of these diseases bear similarities to other microgliopathies (Table 1).

LRRC33 is a leucine-rich repeat-containing protein that can anchor latent transforming growth factor beta-1 (TGF-β1) at the cell surface and is required for the activation of TGF-β1 in macrophages and microglia (Qin et al., 2018) (Fig. 2). In addition, it is a negative regulator of reactive oxygen species (ROS) production, hence the suggested name change to NRROS (Noubade et al., 2014). TGF-β1 is a trophic factor and cytokine that is a key component of highly conserved signaling pathways with broad regulatory roles in metazoan development, including immune modulation (reviewed by Gomes et al., 2005). In brain development, TGF-β1 plays an important role in axon growth and polarity (Yi et al., 2010), regulation of astrocyte morphology, motility, proliferation and differentiation (Diniz et al., 2017), and neuron survival (Brionne et al., 2003; Gomes et al., 2005; Dong et al., 2020). In the CNS, *LRRC33* is primarily expressed in microglia (Wong et al., 2017; Qin et al., 2018).

*Lrrc33* knockout mice show increased CD68^+^ microglia numbers with an altered hypertrophic morphology, along with upregulated cell division and interferon signaling pathways (Wong et al., 2017; Qin et al., 2018). Descriptions of other neuropathological features of these mutant mice show some inconsistencies; whereas one group reported localized demyelination and noted loss of oligodendrocytes (Qin et al., 2018), another reported normal axon numbers and myelination but reactive astrocytes in the CNS (Wong et al., 2017). As conditional deletion of *Lrrc33* in mice at 3 weeks of age causes no neurological effects, it is likely that LRRC33 is required for the establishment of the microglial network during development (Wong et al., 2017). So far, nine individuals with bi-allelic loss-of-function mutations in *LRRC33* have been described, most of whom died between 2 and 4 years of age (Dong et al., 2020; Smith et al., 2020). Patients present with severe white matter defects and neuropathology (Table 1). Pathologically lipid-filled CD68^+^ macrophages lacking a homeostatic microglia gene signature were observed throughout the white matter, particularly in perivascular regions (Smith et al., 2020). Together, this suggests that microglial modulation of TGF-β signaling is key to brain development. Consistent with this, bi-allelic mutations in *TGFB1* itself cause a pediatric neurodevelopmental leukoencephalopathy (Kotlarz et al., 2018).

Ubiquitin specific protease 18 (USP18) is a negative regulator of the type 1 and 3 interferon (IFN-I) pathway by binding to the intracellular domain of interferon alpha and beta receptor subunit 2 (IFNAR2) (Honke et al., 2016; Goldmann et al., 2015) (Fig. 2). Microglia that lack *Usp18* exhibit elevated type I IFN signaling pathway activation (Goldmann et al., 2015). Conversely, overexpression of USP18 suppresses microglial activation by reducing the release of pro-inflammatory cytokines (Xiang et al., 2019). Microglia in *Usp18*-deficient mice show increased phagocytosis of myelin (Schwabenland et al., 2019) and present in clusters in the white matter, strongly resembling the microglial pathology in ALSP and NHD (Goldmann et al., 2015; Schwabenland et al., 2019). These mice exhibit diminished structural white matter integrity (Schwabenland et al., 2019) and low bone density due to osteoclast abnormalities (Yim et al., 2016). Homozygous loss-of-function variants in *USP18* in humans can result in pseudo-TORCH syndrome (PTS), a type of genetic disorder termed a type I interferonopathy, which causes death within days after birth (Meuwissen et al., 2016). A striking hallmark of PTS is defective neuronal migration, partly leading to ectopic neuronal cell bodies in the white matter (Table 1). Immunohistochemistry on patient postmortem brain tissue reveals a robust presence of IBA1^+^ and HLA-DR^+^ microglia throughout the brain (Meuwissen et al., 2016). Additionally, *Usp18*-deficient mice exhibit an elevated number of microglia that appear to have engulfed oligodendrocytes (Schwabenland et al., 2019).

Despite differences in their underlying mechanisms, *LRRC33/NRROS* and *USP18*-related microgliopathies reveal that deficiencies in key microglial immune signaling pathways, in this instance TGF-β and interferon signaling, lead to phagocytic abnormalities in the brain and a consequent reduction in brain integrity and function, with particular involvement of the white matter.

## Primary lysosome or peroxisome dysfunction in leukodystrophies: making a case for microglia

There is strong evidence indicating that microglial involvement in leukodystrophies is caused by lysosomal or peroxisomal defects. Lysosomes are key metabolic hubs for the recycling of macromolecules and metabolites (Platt et al., 2018). Impairments can lead to the accumulation or ‘storage’ of partially undigested material and the reduction of lysosomal function, as a well as a deficit of specific metabolites (Platt et al., 2018). Peroxisomes are membrane-enclosed organelles that contain enzymes involved in lipid metabolism (Titorenko and Rachubinski, 2004). Deficiencies in peroxisomal enzymes can also result in substrate accumulation. It is common that lysosomal and peroxisomal storage disorders present as severe progressive neurodegenerative diseases (Platt et al., 2018). Among these, several are also classified as leukodystrophies (Table 1): MLD, GLD and X-ALD (van der Knaap and Bugiani, 2017).

MLD patients have bi-allelic mutations in the *ARSA* gene that lead to deficiency of arylsulfatase A (ASA), a lysosomal enzyme that digests sulfatides – glycosphingolipids that are highly enriched in myelin sheaths (Eckhardt, 2008; Fig. 2). Accumulation of sulfatides in white matter macrophages, as well as other brain cells including neurons, astrocytes and oligodendrocytes, coincides with severe destruction of the cerebral and deep white matter (Table 1). MLD occurs in up to 2.5 cases per 100,000 individuals in the USA (Platt et al., 2018). Bi-allelic mutations in *GALC*, encoding the lysosomal hydrolase galactosylceramidase, cause Krabbe disease (Suzuki and Suzuki, 1970) (Fig. 2). Galactosylceramidase catabolizes galactosylceramide and galactosylsphingosine (psychosine), both of which are major glycosphingolipids in the myelin membrane (Weinstock et al., 2020). The resulting accumulation of psychosine is thought to underlie the progressive white matter disease (Suzuki, 1998). X-ALD is an X chromosome-linked disorder caused by mutations in the *ABCD1* gene, encoding the ATP-binding cassette (ABC) half-transporter ALDP, resulting in the failed transport of very long-chain fatty acids (VLCFA) into the peroxisome (Kemp et al., 2001) (Fig. 2). VLCFAs aggregate predominantly in the brain and spinal cord via esterification with glycerophospholipids and cholesterol, as these lipids are abundant in the myelin membrane (Schaumburg et al., 1976). This ultimately leads to degeneration of the white matter (Table 1). The estimated incidence of X-ALD is about 1 in 14,700 live births (Bezman et al., 2001; Moser et al., 2016).

The genetic variants defining these leukodystrophies may not immediately appear directly associated with microglia, as the genes are expressed across various brain cell types. However, as microglia are the major phagocytes of the brain, defects in either lysosomes or peroxisomes are likely to substantially affect phagocytosis and waste accumulation in microglia, which could contribute to significant dysfunction. Again, support for microglial involvement in the mechanism of these pathologies is most clearly demonstrated by the beneficial effects of HSCT (reviewed by Musolino et al., 2014; van Rappard et al., 2015). In postmortem brain tissue of MLD patients who received HSCT, active ASA was clearly detectable in macrophages/microglia but not in resident oligodendrocytes and astrocytes, suggesting no cross-correction by healthy donor enzyme. Moreover, transplanted patients had increased numbers of homeostatic macrophages and oligodendrocyte lineage cells, and there was evidence of remyelination (Wolf et al., 2020). Similarly, Weinstock et al. (2020) examined enzyme cross-correction and the effects of HSCT on the peripheral nervous system (PNS) in *Galc*^−/−^ mice and in postmortem spinal cord tissue of GLD patients. In mutant mice, Schwann cells, the myelinating cells of the PNS, require lysosomal Galc to maintain myelin and axonal integrity, and are unable to receive enzyme from the surrounding environment, partly due to ineffective uptake. GLD patients who received HSCT showed an improvement in axonal integrity and myelin thickness, as well as a reduction of phagocytic CD68^+^ microglia, fewer multinucleated phagocyte clumps (also termed globoid cells) and reduced substrate accumulation in macrophages (Weinstock et al., 2020). Although it is important to note that the effects of HSCT can vary tremendously between individuals and between leukodystrophies, and its potential effectivity is limited by the need to treat early and pre-symptomatically (Boucher et al., 2015), these data support the theory that microglia are key players in myelin repair and oligodendrocyte lineage regulation. Importantly, we consider it unlikely that the therapeutic benefit of HSCT in these examples is not linked to an initial contribution of microglial abnormalities to the myelin pathology.

Indeed, the contribution of microglia to the pathology of these leukodystrophies is supported by findings in patient postmortem brain tissue, in which CD68^+^ phagocytic microglia are abundant in both the affected and least-affected white matter of Krabbe disease (Del Bigio, 2018), MLD and X-ALD patient brain tissue (Bergner et al., 2019). Ramified homeostatic microglia numbers decrease continuously towards the demyelinated lesion, until there are only amoeboid phagocytic microglia left (Fig. 1; Bergner et al., 2019; Itoh et al., 2002). Importantly, amoeboid phagocytes show large intracellular phagosomes, waste accumulation and MBP inclusions, indicating disrupted (myelin) phagocytosis (Bergner et al., 2019; Percy et al., 1994). *Galc*^+/−^ mice do no exhibit a neuropathological phenotype in normal circumstances, but upon induced demyelination, microglia show impaired myelin phagocytosis and reduced *Trem2* expression, which is necessary for clearing myelin (Scott-Hewitt et al., 2017). In accordance, patient-derived PNS macrophages show defective myelin degradation, resulting in waste material accumulation (Weinstock et al., 2020). Together, these studies indicate that lysosomal or peroxisomal dysfunction results in waste accumulation in microglia, presumably due to improper degradation, which worsens in the proximity of white matter lesions. Although these diseases likely also have major non-microglial components, the effects seen in microglia appear to be cell-autonomous and not simply responses to pathological processes in other cells of the CNS. This is reminiscent of lysosomal storage disorder phenotypes in zebrafish models (Box 2: The Zebrafish – *In Vivo* Modelling of Glial Cell Dynamics and Myelination in Genetic Brain Disorders), in which abnormal microglia are typically seen in very early embryogenesis, right at the stages at which they seem to shape brain development (Berg et al., 2016; Kuil et al., 2019a; Sanderson et al., 2021).

## Overlapping pathologies indicating possible mechanisms: a perfect storm?

**Fig. 3.**
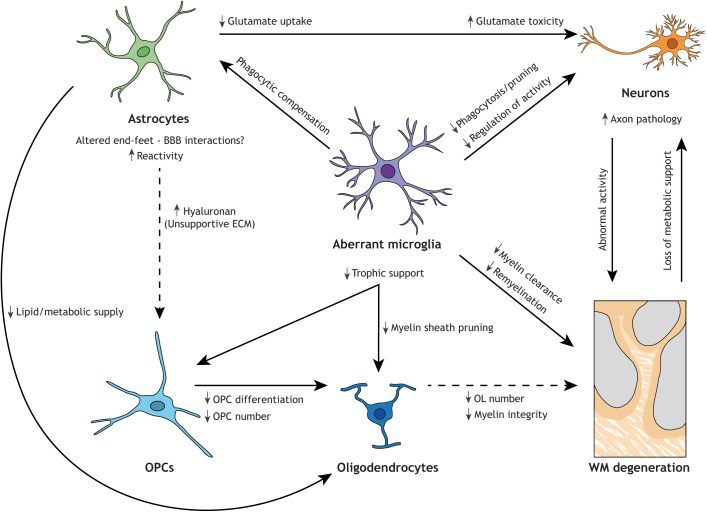
**Loss of microglial functions could lead to white matter degeneration by affecting multiple intercellular connections.** Routes whereby microglia, directly or indirectly, affect the white matter (WM) have been supported by data from experimental studies as described here; however, their relevance to disease remains to be fully explored. Astrocytes compensate for a lack of microglial phagocytosis by becoming more phagocytic, although they are less efficient than microglia and this response may result in the neglect of critical astrocytic functions. Together with the increased astrocytic reactivity observed in leukodystrophies, this can result in disturbed lipid and metabolic supply to oligodendrocytes (OL) and an unsupportive ECM environment for OPCs. Additionally, altered interactions between the BBB and astrocytic end-feet can perturb metabolic supply to brain cells. Aberrant microglia can lead to insufficient trophic support for OPCs and oligodendrocytes, and a diminished oligodendrocyte lineage. Aberrant microglia may also exhibit perturbed clearance and pruning capacity and contribute to impaired remyelination. Both aberrant microglia and affected astrocytes can cause neuronal stress due to neurotoxicity, ineffective phagocytosis and/or dysregulation of neuroactivity. Axonal pathology and abnormal neuronal activation can affect myelination and, in turn, the degeneration of myelin results in a loss of metabolic support for axons. In sum, white matter degeneration in leukodystrophies is likely preceded by distinct effects of aberrant microglia, possibly forming a ‘perfect storm’ of parallel effects particularly detrimental for the myelinated white matter tracts. Solid arrows indicate established interaction/consequence. Dashed arrows indicate hypothesized interaction/consequence in leukodystrophic brain.

Although differences among leukodystrophies do remain profound, we argue that the overlapping pathological features described in this Review are both significant and revealing (Table 1). A detailed examination of these similarities, combined with recent insights from genetic disease models, will aid in understanding the shared underlying mechanisms and cell types involved, which in turn will facilitate the identification of novel targets for treatment. These analyses will also yield important clues about the function of microglia and macrophages in myelin homeostasis and repair in particular, and in maintaining brain health overall. Here, we suggest a variety of possible ways, supported by circumstantial evidence from rare genetic disease, in which aberrant microglia may lead to white matter abnormalities (Fig. 3).

### Axonal pathology

One overlapping hallmark of leukodystrophies is axonal pathology, which may precede white matter degeneration. In fact, a brain biopsy of a patient with ALSP showed abundant spheroids, which are pathological swellings of neuronal processes, 2.5 years before a postmortem examination that revealed fewer spheroids (Marotti et al., 2004). Additionally, the occipital lobe in ALSP patient brains does not show obvious myelin changes but does present axonal spheroids (Jin et al., 2015). Axonal spheroids can be caused by impaired ATP-dependent axonal transport of proteins, lipids, organelles and autophagy-lysosomal degradation, processes that are implicated in several neurological diseases (Sleigh et al., 2019). Other axon pathologies, such as the neuritic beading regularly found in neurodegenerative diseases, are linked to microglial glutamate release leading to inhibition of mitochondrial activity and a rapid drop in neuronal ATP levels (Takeuchi et al., 2005). This energy loss leads to defective axonal transport and potential subsequent excitotoxic neuronal death. Importantly, as myelin provides protection and metabolic support to axons (Aggarwal et al., 2011), myelin degeneration also likely results in axonal defects, making it difficult to pinpoint the initial culprit (Fig. 3).

Axon loss could also occur due to failed axon guidance and growth during development. Defective neuronal migration is observed in *USP18*-related leukodystrophy patients (Meuwissen et al., 2016). In the complete absence of microglia, as in bi-allelic *CSF1R* mutations, there is almost no development of the corpus callosum. This is supported by studies in mouse models showing that microglia are crucial for dorsal axon guidance in the corpus callosum (Pont-Lezica et al., 2014), and for axon guidance and growth in other parts of the brain (Rigby et al., 2020; Squarzoni et al., 2014). However, *Csf1r*-deficient rats have only a slight thinning of the corpus callosum (Keshvari et al., 2020), and there were no apparent commissural abnormalities in zebrafish deficient for *csf1r* (Oosterhof et al., 2018).

Microglia have been shown in mouse studies to prune synapses and axons, which is important for normal brain development (Paolicelli et al., 2011; Schafer et al., 2012). Another intriguing function has been identified in zebrafish, in which neuronal activity steers microglial processes and facilitates direct contact with highly active neurons to reduce stimulus-evoked neuronal excitation (Li et al., 2012). In mice, microglia can also suppress neuronal activity by an adenosine-mediated mechanism (Badimon et al., 2020). Microglia can sense and catabolize the extracellular ATP released by neurons and astrocytes via the purinergic P2YR12 (Badimon et al., 2020). Microglial ablation, or P2RY12 blockade, can induce amplification and synchronization of neuronal excitation, leading to seizures (Badimon et al., 2020). P2RY12-dependent somatic microglia-neuron junctions are found in mouse and human brains, and, upon injury, trigger P2RY12-dependent neuronal protection and regulation of neuronal calcium load (Cserép et al., 2020). Thus, microglial processes can sense, monitor and inhibit neuronal activity and protect neuronal functions. The absence of normally functioning microglia can thereby lead to overexcitation and seizures (Badimon et al., 2020). An as-yet unstudied potential implication of this process is the possibility of aberrant neuronal activity patterns and neuroplasticity in patients with abnormal microglia (Fig. 3). As neuronal activity can regulate and drive myelination, numerous studies, including a recent preprint (Knowles et al., 2020), indicate that these altered activity patterns could subsequently lead to abnormal myelination patterns (Mensch et al., 2015; Marisca et al., 2020; Hughes and Appel, 2020).

### Phagocytic clearance and lipid metabolism

Both NHD and storage-related leukodystrophies show that defective myelin and lipid processing by microglia could be an initiating event for white matter defects. Myelin membranes are incredibly lipid rich, as lipids are required for myelin stability (Schmitt et al., 2015). Hence, disrupted lipid metabolism could cause abnormal membrane lipid composition or the accumulation of toxic lipid species in the brain environment (Schmitt et al., 2015).

Lysosomal and peroxisomal storage-related leukodystrophies have shown that insufficient waste degradation leads to the accumulation of myelin debris and other phagocytic cargo in microglia. During normal aging, the degradative capacity of microglia is known to decrease, leading to lysosomal storage (Safaiyan et al., 2016). Recently, abnormal microglia have been identified in aging white matter in mice. These are characterized by the upregulation of phagocytic activity and lipid metabolism, are found in clusters in the white matter and engage in clearing degenerated myelin (Safaiyan et al., 2021). Dysfunctional myelin recycling by microglia presumably results in excessive myelin debris and (lipid) toxins in the brain environment, which could impede the availability of lipids for myelin formation, and proper clearance of debris is crucial for remyelination (Lampron et al., 2015; Cantuti-Castelvetri et al., 2018). Owing to the high plasticity of developing myelin, which in humans continues into adulthood and is linked to learning and memory (Mount and Monje, 2017; Williamson and Lyons, 2018; Bonetto et al., 2020), white matter could be specifically and devastatingly affected by progressive microglial storage dysfunction. The adult-onset cases of microgliopathies described here could involve an accumulation of detrimental microglial dysfunction throughout life, leading to a progressive inability to maintain white matter integrity. Impairment of myelin degradation, as occurs during normal aging, could be the final blow from where pathology starts to manifest in clinical symptoms.

In addition to clearing myelin debris from the brain environment, microglia also actively phagocytize myelin sheaths during development, a process regulated by neuronal activity. Interestingly, when microglia are ablated, oligodendrocytes maintain excessive myelin sheaths (Hughes and Appel, 2020) and show ultrastructural myelin pathologies, as described in a recent preprint (Djannatian et al., 2021). This suggests that in the absence of healthy functioning microglia, myelin sheaths are not properly pruned during myelin development. One can speculate that improper pruning during development can lead to an unstable myelin structure and integrity (Fig. 3). How and whether this could affect myelin health is not yet fully understood.

### Aberrant astrocytes

Astrocytes characterized by elevated GFAP expression and hypertrophic processes, often termed reactive astrocytes (Box 1; Escartin et al., 2021), are detected in virtually all neurodegenerative diseases, including the leukodystrophies described in this article. Astrogliotic scars are particularly abundant in the most affected white matter lesions in leukodystrophy. Reactive astrocytes can be both pro-inflammatory and neurotoxic, leading to the death of neurons and oligodendrocytes, by losing the ability to promote neuronal survival, synaptogenesis and phagocytosis (Bi et al., 2013; Liddelow and Barres, 2017; Liddelow et al., 2017). However, reactive astrocytes can also have beneficial effects, including limiting tissue damage after injury and modulating the immune response (Faulkner et al., 2004; Myer et al., 2006; Escartin et al., 2021). Hence, it is still unclear whether reactive astrocytes are detrimental to the progression of leukodystrophies or are serving a protective purpose. Similarly, it is unclear what relationship they have to the observed white matter defects.

Two studies have shown that both microglial dysfunction (Konishi et al., 2020) and microglia depletion (Damisah et al., 2020) lead to elevated astrocytic phagocytosis. An enhanced phagocytic phenotype is also observed in reactive astrocytes after brain ischemia (Morizawa et al., 2017). Although astrocytes can phagocytize apoptotic cell bodies and debris, they are much less efficient than microglia (Damisah et al., 2020). When taking over the role of the major phagocyte in the brain in the absence of (functioning) microglia, astrocytes may fail to execute critical astrocytic functions important for maintaining brain health (Fig. 3). For example, lipid and nutrient supply by astrocytes to oligodendrocytes via connexin-coupled gap junctions is essential for proper myelination (Camargo et al., 2017; Orthmann-Murphy et al., 2008). Abnormalities in astrocytic end-feet and blood-brain barrier (BBB) defects have also been noted in neurodegenerative diseases (Oksanen et al., 2019). The surrounding of vessels by astrocytic end-feet is vital for BBB integrity and overall brain health (Box 1). Compromised BBB integrity could contribute to the calcifications observed in most microglia-related leukodystrophy patients (Table 1). Notably, the BBB appears to be intact in *Csf1r*^−/−^ rats (Pridans et al., 2018), and vascular barriers may even be less permissive. Further research is needed to establish whether and how astrocytes lose certain functions vital for white matter and overall brain health in the absence of functioning microglia.

Important clues on the impact of aberrant astrocytes on white matter integrity come from astrocyte-related leukodystrophies. Gain-of-function mutations in *GFAP* lead to Alexander disease (AxD), which is characterized by a severe reactive astrocyte phenotype, including hypertrophic GFAP^+^ processes and astrocytic cytoplasmic inclusions of GFAP called Rosenthal fibers (Sosunov et al., 2018). AxD mouse models have revealed reduced glutamate transporter expression on astrocytes, indicating decreased glutamate uptake by astrocytes (Tian et al., 2010; Hagemann et al., 2009), which is supported by an observed glutamate toxicity-induced neuron loss in a *Drosophila* AxD model (Wang et al., 2011). Astrocytes remove ∼90% of all released glutamate in the brain, thereby maintaining glutamate homeostasis by regulating the balance between glutamate uptake and release in the synaptic cleft (Mahmoud et al., 2019). Excessive glutamate toxicity is hypothesized to be one of the mechanisms driving myelin loss in AxD (Sosunov et al., 2018). As elevated GFAP^+^ astrocytes are also abundantly present in microglia-related leukodystrophies, and glutamate toxicity is a known cause of axon pathology, a similar astrocyte phenotype could partly underlie the white matter defects in microglia-related leukodystrophies (Fig. 3). Deposition of the glycosaminoglycan hyaluronan in the ECM, which occurs in the astrogliosis seen in various diseases and in AxD mice, has also been suggested to contribute to myelin defects (Sosunov et al., 2018). Hyaluronan inhibits oligodendrocyte precursor cell (OPC) maturation (Box 1, Fig. 3) and is thought to be important to the pathology of vanishing white matter disease, another astrocyte-related leukodystrophy (Sosunov et al., 2018). Microglia engulf the ECM in an IL-33-dependent manner to promote synapse plasticity and deficiency of this process leads to an accumulation of ECM proteins around synapses and dendrites (Nguyen et al., 2020). Hence, disturbed white matter integrity due to abnormalities in ECM composition, resulting from the inability of microglia to properly prune and maintain it, is a promising future line of investigation.

### The zebrafish: a promising leukodystrophy model

Looking forward, it will be important to combine and compare data from diverse models, human material and clinical observations of disease to improve insight into the cellular brain. Rare brain diseases can give unique and precious clues into human genetics, physiology and pathology. Nevertheless, high quality postmortem brain tissue is very rare, and studies often allow only limited quantitative analysis. So far, the majority of available leukodystrophy animal models have been mice. Hence, their neuropathological and clinical features are thoroughly characterized. Mouse models allow thorough investigation by highly standardized techniques and reagents in a well-characterized system. Yet, despite the substantial advantages of mouse models, direct *in vivo* cell biological observations are still challenging, and developmental processes in particular are relatively obscured. Cerebral organoids derived from iPSCs are improving but are not yet able to effectively model myelination and the native interactions of multiple glial cell types. The zebrafish has repeatedly proven its value as a system for unparalleled developmental observations of *in vivo* cellular mechanisms, and its many unique advantages readily complement data obtained through other model systems (Box 2). Especially for understanding the highly interactive cellular processes of myelination and glial cell development in leukodystrophy, the ability to perform real-time, unbiased and non-invasive imaging of dynamic cellular interactions during early embryonic development in a whole organism makes the zebrafish a promising model that can take the field far beyond the current knowledge (Rutherford and Hamilton, 2019; Keefe et al., 2020). The major players in leukodystrophies have been well-characterized in zebrafish (Herbomel et al., 1999; Peri and Nüsslein-Volhard, 2008; Oosterhof et al., 2017; Marisca et al., 2020; Park et al., 2002; Mu et al., 2019; Chen et al., 2020) and have led to major discoveries in the functions of glial cells and myelination (Mensch et al., 2015; Almeida et al., 2018; Djannatian et al., 2019; Hughes and Appel, 2019, 2020; Marisca et al., 2020; Mu et al., 2019; Li et al., 2012). Indeed, a number of genetic zebrafish models have provided important data on the disease mechanisms of several leukodystrophies (Pant et al., 2019; Strachan et al., 2017), including the involvement of microglia in the early disease pathology of *CSF1R*-related leukodystrophy (Oosterhof et al., 2018, 2019) and *RNASET2*-related leukodystrophy (Haud et al., 2011; Hamilton et al., 2020; Weber et al., 2020), and the identification of possible therapeutic targets for the treatment of X-ALD (Raas et al., 2021).

### Conclusions

Leukodystrophies involving microglia raise intriguing hypotheses regarding the impact of macrophages and microglia on the developing and adult brain, in particular on myelination. In addition, they may help provide insight regarding the issues of optimal macrophage and microglia enhancement or depletion strategies that, although avoiding detrimental side effects related to macrophage function, could treat human disease. It is now clear that depleting microglia, at least temporarily via CSF1R inhibitors, can be beneficial in mouse models of Alzheimer's disease, and therefore has clinical potential. Conversely, studies of rare human disease complemented by zebrafish and rodent disease models has demonstrated that there are undoubtedly negative effects associated with microglial depletion. Therefore, understanding the breadth of roles microglia have in normal human brain development and in the maintenance of adult brain health would be very helpful to further fine-tune methods of modulating microglia in disease to mitigate disease progression and benefit recovery. Insight from *CSF1R*-related leukodystrophies and CSF1R-deficient animal models could assist in deducing the consequences of the absence or local depletion of macrophages and microglia on the human brain.

It is clear that several important questions remain to be answered regarding how microglia, as well as peripheral macrophages, affect myelin development and the maintenance of white matter and brain health in both development and adult life. For example, defective osteoclasts and other macrophage populations can lead to skeletal abnormalities, as observed in some of the leukodystrophies described here. Skeletal abnormalities are present in several microglia-related leukodystrophies and are likely caused by abnormal osteoclast populations, which originate from similar hematopoietic progenitors as other macrophages and microglia (Bar-Shavit, 2007). In the case of bi-allelic loss of *CSF1R* function, skeletal abnormalities are identified across vertebrate model systems. It is unclear whether this bone phenotype could contribute to the neuropathology, opening up intriguing opportunities for future research.

Integrating the application of novel methods on postmortem brain tissue of patients, including single-cell and high-throughput genomic, proteomic and metabolomic approaches with genetic model systems, in particular the zebrafish, will aid in unraveling how both microglia and other brain cell types modulate white matter integrity and overall brain health. Importantly, rare genetic brain disorders provide a unique window into human brain physiology and continue to provide clues to the inner workings of the brain and to potential avenues of therapeutic modulation that may be pursued for a broad range of neurological diseases.
